# Efficiency of a microplastic removal system from synthetic wastewater using a chemical process combined with simple filtration

**DOI:** 10.1038/s41598-026-59084-8

**Published:** 2026-06-20

**Authors:** Poonyaporn Poolborwornrak, Santisith Khiewkhern, Jindawan Wibuloutai, Prapat Pentamwa

**Affiliations:** 1 Faculty of Public Health, Mahasarakham University, Maha Sarakham, 44150 Thailand; 2 Institute of Public Health, Suranaree University of Technology, Nakhon Ratchasima, 30000 Thailand

**Keywords:** Efficiency, Microplastics, Synthetic wastewater, Chemical process, Filtration, Chemistry, Engineering, Environmental sciences, Water resources

## Abstract

Microplastic contamination in freshwater ecosystems poses a critical environmental challenge, necessitating effective removal technologies in wastewater treatment. This research aims to evaluate the efficiency of a hybrid removal system for polyethylene microplastics (particle sizes 0.15–1 mm) by integrating chemical coagulation with simple sand filtration. The performance of three coagulants—alum, polyaluminium chloride (PAC), and ferric chloride—supplemented with chitosan as a coagulant aid, was optimized across various dosages and pH levels, followed by the evaluation of a sand filter column under different flow rates. The results indicated that coagulation efficacy was pH-dependent, peaking at pH 7, where PAC achieved the highest removal efficiency of 77.41 ± 4.47% for fine 0.15 mm particles, followed by alum (73.22 ± 3.54%) and ferric chloride (63.18 ± 3.63%); notably, the combined system operating at a flow rate of 15 mL/min (Experimental Set 1) attained an exceptional overall removal efficiency of 99.93%. These findings demonstrate that coupling chemical coagulation with low-cost sand filtration offers a robust and scalable solution for significantly reducing microplastic discharge in synthetic wastewater applications.

**Supplementary Information:**

The online version contains supplementary material available at https://doi.org/10.1038/s41598-026-59084-8.

## Introduction

Plastics have been a part of human daily life for a long time. They are widely used with a growing trend of increasing consumption in items like plastic bags, packaging, and furniture. After use, these items become plastic waste, and when managed improperly, they cause environmental pollution^1^. Microplastics are plastics smaller than 5 millimeters, which are lightweight and highly persistent in the environment^2^. These plastics degrade when exposed to environmental factors and various processes^3^, such as photodegradation, thermo-oxidative degradation, and biodegradation^4^. Because of their small size, microplastics are found widely in both water and sediment^5^ leading to their transfer to marine life and accumulation in the food chain^6^. As a result, humans have the potential to ingest microplastics by consuming contaminated aquatic animals, which may have adverse health effects^7^.

Numerous research reports on the contamination, spread, and severe impact of microplastics on freshwater ecosystems, marine ecosystems, terrestrial ecosystems, humans, wildlife, freshwater, and marine animals, as well as drinking water and tap water systems^7–10^. Freshwater ecosystems, in particular, show a growing trend of microplastic contamination from the discharge of wastewater, household and urban waste, and wastewater treatment plants, making them a significant pathway for microplastics to enter the environment. Some microplastics that can float in water are discharged with treated water into natural water sources, leading to environmental contamination^11^.

Previous studies have assessed microplastic removal efficiency in varying locations; Van Do et al. (2022) investigate microplastic contamination in three wastewater treatment plants in Da Nang, Vietnam, microplastics in the effluents ranged from 138 to 340 particlesperliter^12^. Tadsuwan et al. (2022) examined microplastic removal in a conventional wastewater treatment system in Thailand. Samples were collected from return activated sludge, and the results showed that microplastics in the final effluent were 10.67 ± 3.51 particlesperliter^13^. Furthermore, Sturm et al. (2025) compared microplastic levels in the effluents of three municipal wastewater treatment plants employing different treatment processes. The study reported 21.8 particles/L in the effluent of a conventional three-stage WWTP, while 15.1 particles/L were found in both the four-stage WWTP and the MBR system^14^. This data highlights the release of microplastics into the environment. Although various advanced wastewater treatment technologies have been developed, such as membrane processes, advanced filtration, and advanced oxidation processes, coagulation remains one of the most widely used methods due to its operational simplicity, rapid implementation, and high efficiency in removing suspended particles from water^15^. In the context of microplastic removal, which involves small and stable particles in aqueous environments, coagulation and flocculation have been applied as fundamental processes to reduce microplastic concentrations in wastewater treatment systems^16,17^. These processes destabilize colloidal and fine particles through charge neutralization and sweep flocculation by metal hydroxide precipitates, resulting in the formation of larger flocs that can settle more easily^18^. This mechanism is particularly important for microplastic removal, as most microplastics carry negative surface charges and have densities close to that of water, making them difficult to settle under normal conditions. Coagulants used in wastewater treatment and microplastic removal can be classified into two main groups: inorganic coagulants and organic coagulants. Among inorganic coagulants, aluminum- and iron-based compounds such as aluminum sulfate, ferric chloride, and poly-aluminum chloride are widely used in conventional wastewater treatment systems^16,19^. Studies have reported that these coagulants can significantly remove microplastics, particularly microbeads and small-sized particles, which tend to be effectively captured and incorporated into metal hydroxide flocs^19^. On the other hand, organic coagulants and coagulant aids, such as polyacrylamide, polyDADMAC, polyamines, and chitosan, play an important role in enhancing flocculation efficiency. Their primary mechanism is polymer bridging, in which high–molecular weight polymer chains adsorb onto multiple particles simultaneously, forming larger, stronger flocs with higher settling velocities^18^. Furthermore, microplastic research has indicated that the addition of cationic polymers, such as cationic PAM, in combination with aluminum-based coagulants can significantly improve microplastic removal efficiency, particularly for small, low-density particles that are otherwise difficult to settle^19^.

A previous study Ma et al. (2019) reported that the use of ferric chloride (FeCl_3_) as a coagulant in combination with polyacrylamide (PAM) as a coagulant aid achieved a removal efficiency of 45.34 ± 3.93% for polyethylene microplastics^19^. Another study demonstrated Shahi et al. (2020) that alum used as a coagulant removed 70.7% of polyethylene microplastics^20^. Furthermore, a study Zhou et al. (2021) showed that the coagulation process promotes the aggregation of small plastic particles into larger, more readily settleable flocs, resulting in removal efficiencies of 99% for particle sizes of 0.5–1 mm and 98% for sizes of 1–5 mm^21^. In general, coagulants can be broadly classified into synthetic and natural types. Synthetic coagulants like Al₂(SO₄)₃ and FeCl_3_ and polymeric coagulants such as polyamines, Polydiallyldimethylammonium chloride, and polyacrylamide are often used in water and wastewater treatment^22^. While synthetic coagulants are highly effective, they have significant drawbacks, including metal and organic residues in the treated water and the generation of large quantities of non-biodegradable sludge, which can negatively impact the environment^23^. The exploration of natural coagulants has gained significant interest recently. Natural coagulants can be sourced from plants, animals, and microorganisms and contain coagulating agents like proteins and polysaccharides, such as starch, pectin, sodium alginate, gum, and chitosan^24^. These have been studied as potential coagulants and coagulant aids for microplastic removal. Several researchers have reported using chitosan in conjunction with coagulants. Raj et al. (2024) reported on the coagulation of polystyrene microplastics using FeCl_3_ with chitosan^25^, while He et al. (2024) and Huang et al. (2023) used polyaluminium chloride with chitosan for the coagulation of polyethylene terephthalate^26,27^. However, microplastics found in water sources are of various types^28^, and the types of coagulants used also vary, which leads to different microplastic removal efficiencies^19^.

Therefore, the objective of this study is to investigate the efficiency of a microplastic removal system from synthetic wastewater using a chemical process combined with simple filtration. This research covers the study of the chemical properties of three types of coagulants alum, polyaluminium chloride, and ferric chloride and the coagulant aid, chitosan. It also evaluates the efficiency of simple filtration for removing microplastics from synthetic wastewater. The study is divided into two main experiments: determining the optimal conditions to enhance the efficiency of the coagulation process and studying the efficiency of filtration for microplastic removal. The findings will serve as a guideline for preventing and solving problems caused by plastic waste, as well as for developing technology for water quality control plants to further reduce the spread of microplastics into natural water sources.

## Experimental method

This study investigated the efficiency of a microplastic removal system for synthetic wastewater using chemical treatment combined with simple filtration, employing coagulants and flocculants together with a filtration process. The study was divided into two experiments.

### Evaluation of microplastic removal efficiency using the chemical coagulation process

Polyethylene microplastics were selected as the test material. The plastic samples were mechanically ground and sieved to obtain particle sizes of 0.15, 0.3, 0.5, and 1 mm. Chemical coagulation experiments were conducted using a Jar Test apparatus. The coagulants tested included alum, polyaluminium chloride, and ferric chloride, while chitosan was used as the flocculant. The experimental design included coagulant dosages of 30, 60, 90, 120, and 150 mg/L, with a fixed flocculant dosage of 20 mg/L.

### Evaluation of microplastic removal efficiency of filtration media

Filtration experiments were conducted using a laboratory-scale glass sand filtration column. The experiment was divided into three experimental setups and performed using distilled water spiked with polyethylene microplastics of sizes 0.15, 0.3, 0.5, and 1 mm. The filtration media consisted of gravel and sand of six size fractions, including gravel of 5 mm and > 5 mm, and sand with particle sizes of 0.15, 0.3, 0.5, and 1 mm. The system was operated while intermittently mixing the prepared sample tank, after which the water was introduced into the sand filtration column at flow rates of 15, 30, and 45 mL/min.

**Fig. 1:**
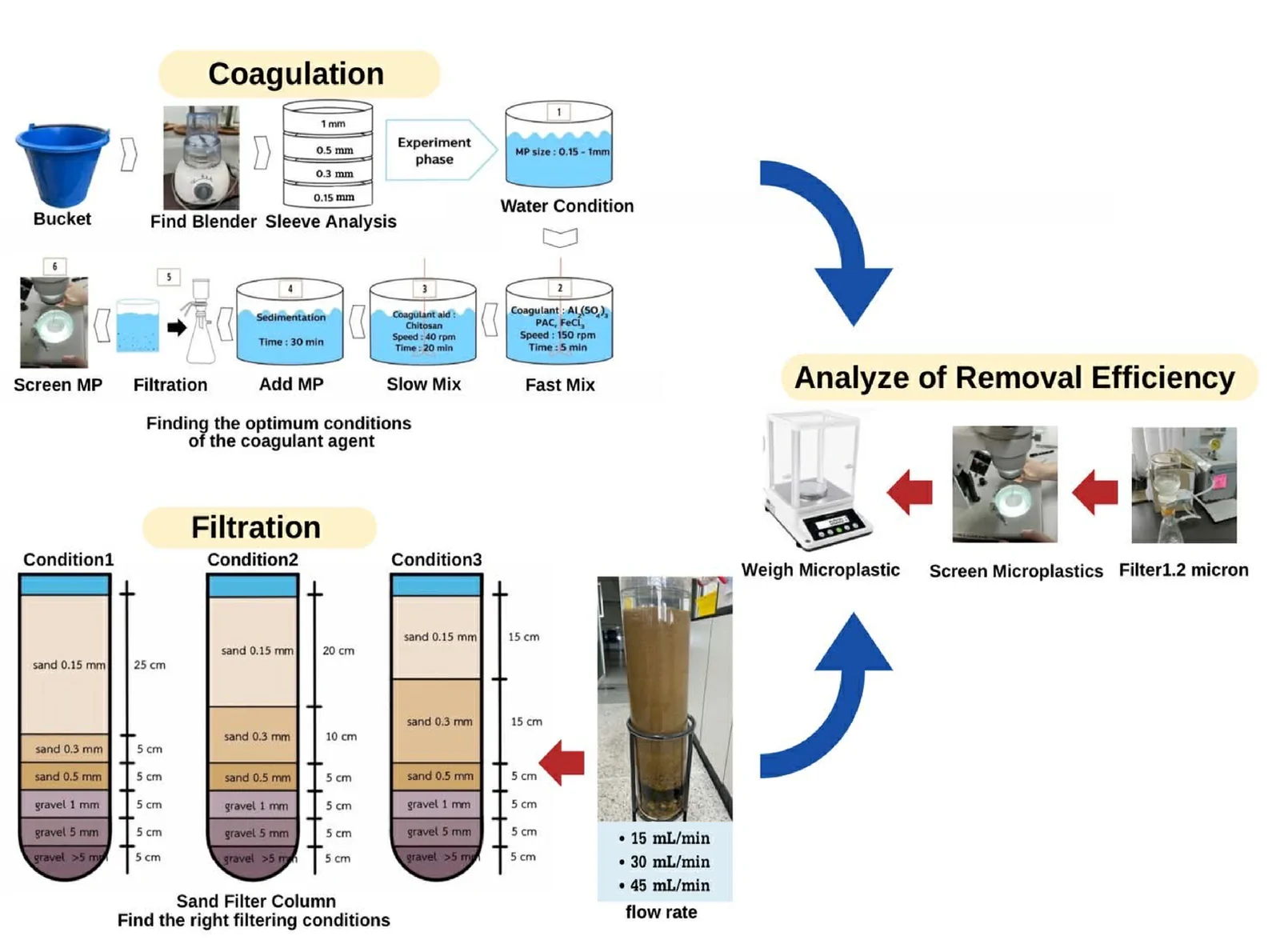
Schematic diagram of the microplastic removal experiments.

After completion of the coagulation and filtration processes, the effluent samples were filtered using a vacuum filtration system with 1.2 μm pore-size filter paper. The filters were dried in a hot air oven at 105 °C for 12 h, placed in a desiccator, and the collected microplastics were weighed using an analytical balance with a precision of five decimal places to calculate the microplastic removal efficiency. The study procedures are detailed as shown in Fig. 1.

### The testing microplastic removal efficiency using a chemical coagulation process

The plastic samples were cut into small pieces, then ground and size-fractionated using sieve analysis into size ranges of 0.15, 0.3, 0.5, and 1 mm. Experiments were conducted using 1000 mL of distilled water spiked with 0.1 g of polyethylene microplastics. Coagulants, including alum, polyaluminium chloride, and ferric chloride (FeCl_3_), were then added at dosages of 30, 60, 90, 120, and 150 mg/L, along with a coagulant aid (chitosan) at 20 mg/L. The mixture was rapidly mixed at 150 rpm for 5 min, followed by slow mixing at 40 rpm for 20 min, and allowed to settle for 30 min. After coagulation and settling, the samples were filtered using a vacuum filtration system, and the retained microplastics were weighed using a five-decimal-place analytical balance to calculate the microplastic removal efficiency. The study procedures are detailed as shown in Fig. 1.

#### Microplastic sample selection

The microplastic samples for this study were sourced from commercially available plastics. Polyethylene, specifically from plastic water containers, was selected because it is one of the top three most common types of plastic found contaminating community wastewater sources. A study that sampled wastewater from the municipal wastewater treatment plant in Chaiyaphum, Thailand, found that polyethylene, polypropylene, and polyester polymers accounted for 45%, 35%, and 20% of the total, respectively. The study by Van Do et al. (2022) identified the polymers polyethylene terephthalate, polyethylene, nylon, and polyvinyl chloride, respectively^12^. Meanwhile, Tadsuwan et al. (2022) reported the presence of polyethylene terephthalate, polyethylene, and polypropylene, respectively^13^. Therefore, polyethylene microplastics were chosen as the sample for this study.

#### Preparation of microplastic samples

Plastic samples were cut into small pieces and then ground using a Sharp EM-ICE Power grinder. The ground plastic samples were then separated by size using the sieve analysis method (ASTM, 2004). The sieve opening sizes used in this study followed ASTM standards. The specific microplastic sizes chosen for this study were based on findings from previous research. Wang et al. (2018) found that microplastic particles detected in community wastewater were in the size range of 0.3–1.0 mm^29^, while, Tadsuwan et al. (2022) reported that microplastics in the effluent were found within the size range of 0.05–0.5 mm^13^. Therefore, for this study, plastic samples were selected with sizes corresponding to sieve openings of 0.15, 0.3, 0.5 and 1 mm.

#### Preparation of coagulant and coagulant aid concentrations

The coagulants used in this study were alum, polyaluminium chloride, and ferric chloride. They were prepared by dissolving 2 g of each in distilled water and adjusting the volume to 1000 mL. The coagulant aid used in this study was chitosan. It was prepared by dissolving 1 g of chitosan in distilled water and adjusting the volume to 1000 mL.

### Coagulation process

The chemical coagulation process in this study was conducted using a Wizard PLUS 6 LED Jar Test apparatus, which has 6 paddles. For each experiment, 1000 mL of distilled water was added. The rapid mixing condition was set to 150 rpm for 5 min, followed by a slow mixing condition at 40 rpm for 20 min. The sedimentation time was 30 min. The microplastic concentration was set at 0.1 g per 1000 mL of water, as shown in Fig. 1.

#### Study of optimal coagulant conditions for microplastic removal

To determine the optimal coagulant dosage, 1000 mL of distilled water was measured, and a coagulant (alum, polyaluminium chloride, and ferric chloride) was added. The coagulant dosages studied were 30, 60, 90, 120, and 150 mg/L, respectively. The coagulant aid was added at a concentration of 20 mg/L during the slow mixing stage. Each experiment was performed in triplicate. The selected concentration range of coagulants in this study was derived from a systematic literature review. The study by Azizi et al. (2023) applied coagulant dosages in the range of 8–140 mg/L and reported that 60, 86, and 140 mg/L achieved significant microplastic removal efficiencies. Specifically, PAC 60 mg/L, AlCl₃ 140 mg/L, and FeCl_3_ 60–112 mg/L were applied at relatively high dosages to enhance effective particle aggregation^30^. Similarly, Eamrat et al. (2024) investigated alum as a coagulant within a concentration range of 10–200 mg/L and found that the optimal condition was 20 mg/L when combined with 1 mg/L of chitosan^31^. Moreover, Zhou et al. reported that using PAC and FeCl_3_ at 90 mg/L resulted in polyethylene removal efficiencies of 29.7% and 17%, respectively^21^. This is consistent with the findings of Luu et al. (2025) who applied aluminum chloride and ferric chloride within a dosage range of 0–120 mg/L and identified 90 mg/L as the optimal condition, achieving microplastic removal efficiencies of 91.7% and 98.3%, respectively^32^.

#### Study of optimal pH for microplastic removal

1000 mL of distilled water was measured, and the optimal amount of each coagulant (alum, polyaluminium chloride, or ferric chloride), as determined from the previous experiment, was added. The pH levels studied ranged from 4 to 9. The optimal pH was determined by analyzing the microplastic removal efficiency of the water samples after the experiment.

### Testing the microplastic removal efficiency of the filtration medium

This study investigated a microplastic removal method by modifying the Rapid Sand Filter (RSF) method from Talvitie et al. (2017) to evaluate the efficiency of a water filtration medium in removing microplastics^33^.

#### Materials used in the filtration process experiment

Glass Filter Column: The glass columns used were 3 mm thick with a diameter of 14.5 cm and a height of 60 cm. Filtration Medium: The filtration medium consisted of gravel and sand in six different sizes: gravel > 5 mm and 5 mm, and sand at 1 mm, 0.5 mm, 0.3 mm, and 0.15 mm. For all three experimental setups, the gravel layers were set at a thickness of 5 cm. The thicknesses of the 0.3 mm and 0.15 mm sand layers were determined.

#### The design of the filtration process experiment

The experiment was conducted using three distinct glass sand filters, with each setup run in triplicate. The system was operated to test the efficiency of the filtration medium according to the following steps:

The experiment used distilled water mixed with polyethylene microplastics of sizes 0.15, 0.3, 0.5 and 1 mm, which were obtained by grinding plastic into small fragments. The ratio was 0.3 g of microplastics per 3 L of water.

The glass sand filter column system was started. The water sample in the preparation tank was stirred periodically. The water was then allowed to flow into the glass filter column at flow rates of 15 ml/min, 30 ml/min, and 45 ml/min, as shown in Fig. 1.

#### Calculation of microplastic removal efficiency

This step of the study involves calculating the microplastic removal efficiency after the experimental setups have completed the coagulation and filtration processes. The samples were filtered using a vacuum filter with a 1.2-micron pore size filter paper. The filtered samples were then placed in a hot air oven at 105 °C for 12 h to dry, followed by a desiccator. The resulting microplastic samples were weighed using a five-decimal-place balance to calculate the removal efficiency. The microplastic (MP) removal efficiency is therefore calculated as follows:

$$\% {\mathrm{Removal}} = \frac{{{\mathrm{Initial}}\;{\mathrm{microplastics}}\left( {\mathrm{g}} \right) - {\mathrm{microplastics}}\;{\mathrm{after}}\;{\mathrm{coagulation}}\left( {\mathrm{g}} \right)}}{{{\mathrm{Initial}}\;{\mathrm{microplastics}}\left( {\mathrm{g}} \right)}} \times 100$$

#### Experimental quality control

Quality control in the laboratory experiments was carefully implemented to minimize sample contamination at every stage of the analysis. Prior to sample preparation, contamination control measures were applied by sealing the laboratory room and operating an air purifier for 30 min to reduce airborne microplastic contamination. The use of plastic equipment was avoided as much as possible, and glassware was used instead. All glassware was thoroughly cleaned before use: first washed with distilled water and 99.00% ethanol, followed by three rinses with distilled water to prevent any additional contamination during analysis. Laboratory benches were cleaned before each experiment, and researchers wore gloves and laboratory coats throughout the study. All experimental equipment was washed with detergent and then rinsed twice with distilled water prior to use. In addition, all experiments were conducted in restricted-access areas, and all samples were covered with aluminum foil to prevent airborne contamination.

### Statistical analysis

Descriptive statistics, including the mean, standard deviation, and percentage, were used to report the research results. Comparative analyses were conducted to examine differences in microplastic removal efficiency across optimal factors (coagulant dose and pH) for each coagulant in the water samples.

## Results and discussion

### Study of optimal coagulant conditions for microplastic removal efficiency

#### Study of optimal alum conditions

**Fig. 2:**
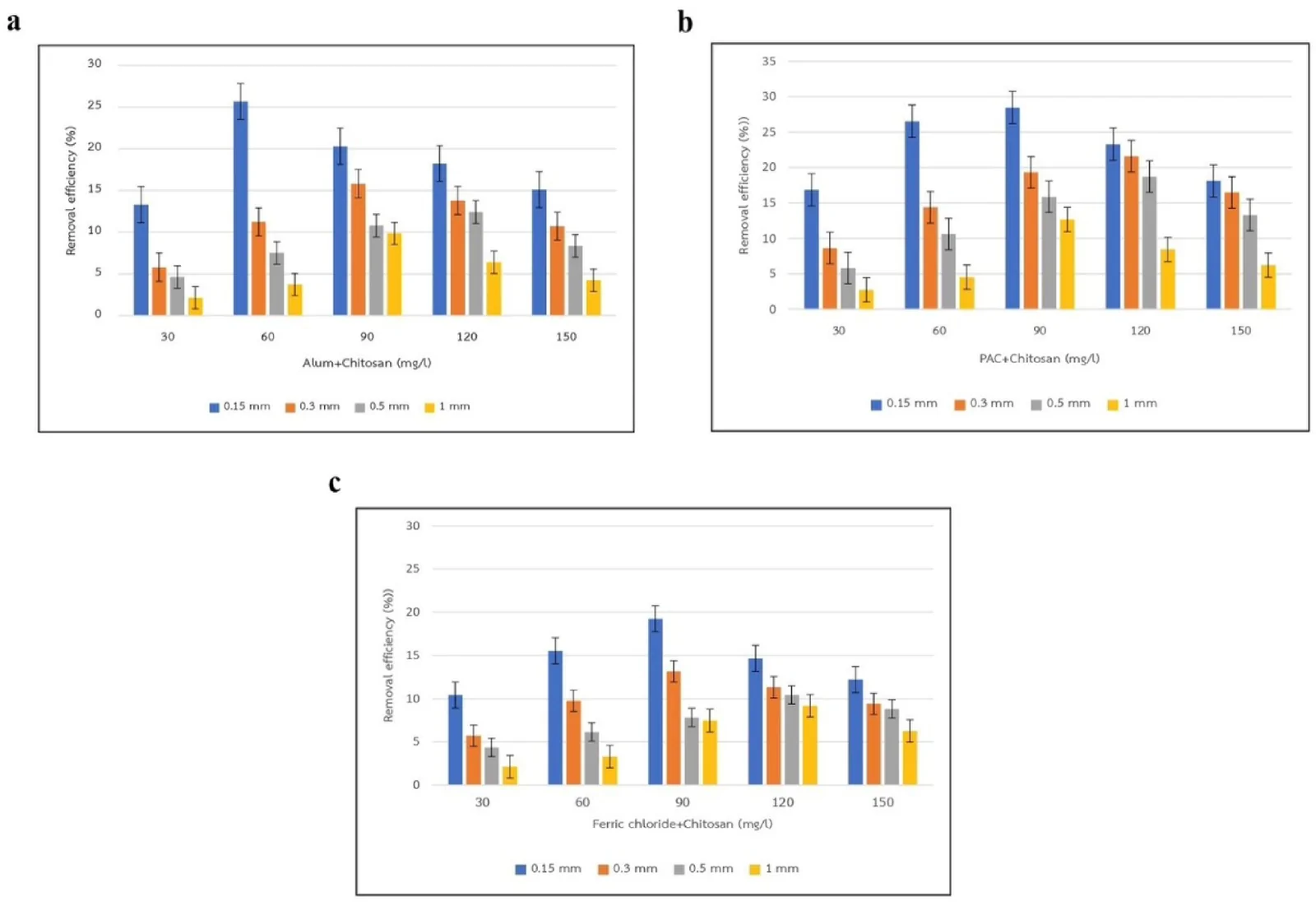
Effect of coagulant dosage on the percentage removal of microplastics with sizes of 0.15, 0.3, 0.5 and 1 mm. (**a**) Alum, (**b**) PAC, (**c**) Ferric Chloride.

The study investigated the effect of alum concentration on microplastic removal efficiency at concentrations between 30 and 150 mg/L. The results, shown in Fig. 2a, indicate that a concentration of 60 mg/L increased the removal efficiency for the 0.15 mm size from 13.33% to 25.68%. At a concentration of 90 mg/L, the removal efficiency decreased to 20.32%. A concentration of 90 mg/L increased the removal efficiency for 0.3 mm and 1 mm sizes from 5.82% to 2.15% to 15.84% and 9.86%, respectively. At a concentration of 120 mg/L, the removal efficiency for these sizes decreased to 13.81% and 4.25%. A concentration of 120 mg/L increased the removal efficiency for the 0.5 mm size from 4.64% to 12.44%. At a concentration of 150 mg/L, the removal efficiency for this size decreased to 8.37%.

#### Study of optimal polyaluminium chloride conditions

The study investigated the effect of polyaluminium chloride concentration on microplastic removal efficiency at concentrations between 30 and 150 mg/L. The results, shown in Fig. 2b, indicate that a concentration of 90 mg/L increased the removal efficiency for the 0.15 mm and 1 mm sizes from 16.85% to 2.73% to 24.48% and 12.66%, respectively. At a concentration of 120 mg/L, the removal efficiency for these sizes decreased to 23.30% and 8.44%. A concentration of 120 mg/L increased the removal efficiency for the 0.3 mm and 0.5 mm sizes from 8.64% to 5.80% to 21.61% and 18.73%, respectively. At a concentration of 150 mg/L, the removal efficiency for these sizes decreased to 16.50% and 13.31%.

#### Study of optimal ferric chloride conditions

The study investigated the effect of ferric chloride concentration on microplastic removal efficiency at concentrations between 30 and 150 mg/L. The results, shown in Fig. 2c, indicate that a concentration of 90 mg/L increased the removal efficiency for the 0.15 mm and 0.3 mm sizes from 10.41% to 5.72% to 19.24% and 13.15%, respectively. At a concentration of 120 mg/L, the removal efficiency for the 0.3 mm size decreased to 14.64%. A concentration of 120 mg/L increased the removal efficiency for the 0.5 mm and 1 mm sizes from 4.36% to 2.13% to 10.44% and 9.18%, respectively. At a concentration of 150 mg/L, the removal efficiency for these sizes decreased to 8.81% and 6.26%.

#### Results of optimal coagulant conditions for microplastic removal

When comparing the coagulant concentrations of the three types, alum, polyaluminium chloride (PAC), and ferric chloride were most effective at removing 0.15 mm microplastics, with removal efficiencies of 25.68%, 28.48%, and 19.24%, respectively. 1 mm microplastics had the lowest removal efficiency at 9.86%, 12.66%, and 9.18%, respectively. This aligns with the findings of Lapointe et al. (2020) and Ma et al. (2019), who studied the effect of polyethylene microplastic size (< 0.5–5 mm) on removal efficiency using coagulation with FeCl₃⋅6 H₂O and AlCl_3_⋅6 H_2_O as coagulants^34,19^. They found that removal efficiency decreased as particle size increased. PE particles sized 0.5–5 mm had low removal efficiency (18.34 ± 3.28%), while particles smaller than 0.5 mm had the highest efficiency (45.34 ± 3.93%). Similarly, Eamrat et al. (2024) studied the use of alum as a coagulant and chitosan as a coagulant aid for polyethylene of sizes 1, 0.3, and < 0.090 mm. They found that the < 0.090 mm size had the highest removal efficiency at 87.75 ± 3.89%, while the 1 mm and 0.3 mm sizes had lower efficiencies at 1.80 ± 0.42% and 21.25 ± 1.06%, respectively^31^. This variation may be because the particles were larger than the flocs composed of aluminum or chitosan. In the case of alum, sweep flocculation is often observed with large particles and high alum doses (20–50 mg/L), whereas charge neutralization is typically seen with small particles and low alum doses (10 mg/L). The efficiency of using coagulants in combination with flocculants for microplastic removal has been extensively studied, and it has been found that combined use results in greater reduction or removal of microplastics compared to using a single coagulant^35,19^. In this study, alum, polyaluminum chloride, and ferric chloride were used, and the optimal dosages were determined to be 60 mg/L, 90 mg/L, and 90 mg/L, respectively. Alum and PAC provided comparable maximum removal efficiencies. The results indicate that smaller microplastics exhibit higher removal efficiency through chemical processes compared to larger ones. Therefore, if wastewater treatment systems contain large quantities of larger microplastics, additional treatment processes such as sand filtration or membrane filtration are required to enhance the removal efficiency prior to discharge into the environment. This is essential for protecting ecosystems and living organisms, as well as reducing health risks associated with the consumption or use of food and drinking water contaminated with microplastics. However, measurement methods for microplastics vary across studies, and currently, there is no internationally standardized method for analysis procedures, sampling, or appropriate detection techniques, which may contribute to the variability of microplastic removal results reported in different studies.

#### Study of optimal coagulant pH for microplastic removal

**Fig. 3:**
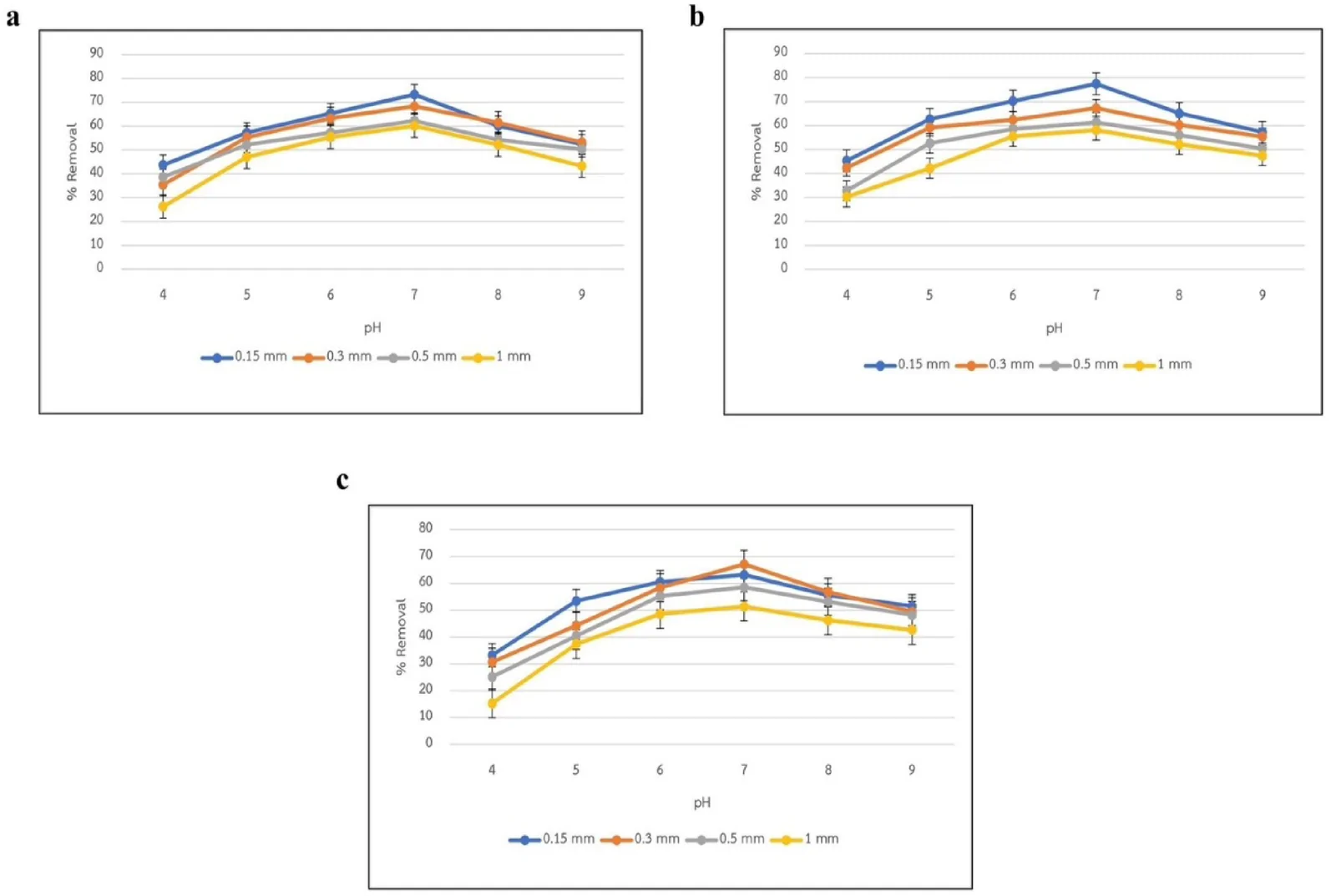
The effect of pH on the percentage of microplastic turbidity removal for sizes of 0.15, 0.3, 0.5 and 1 mm. (**a**) Alum, (**b**) PAC, (**c**) Ferric Chloride.

The turbidity of the solution was measured before and after coagulation to determine the microplastic concentration in the water. The removal percentage was calculated using the measured turbidity values. From Fig. 3a and b, at a pH of 7, alum and polyaluminium chloride showed the highest efficiency for removing turbidity of 0.15 mm microplastics, achieving 73.22% and 77.41% removal, respectively. From Fig. 3c, at a pH of 7, ferric chloride had the highest efficiency for removing turbidity of 0.3 mm microplastics, reaching 67.12%. As the pH increased, the removal efficiency decreased.

#### Study of optimal coagulant pH for microplastic removal

This study investigated the effect of initial pH levels ranging from 4 to 9. A comparison of the optimal pH for the three coagulants revealed that a pH of 7 was the most suitable for coagulation. As the pH increased beyond this point, the removal efficiency decreased. In this experiment, chitosan was used as a coagulant aid. At pH levels above 8, chitosan becomes negatively charged^36^, which leads to colloid stabilization and reduced removal efficiency. PE MPs are negatively charged across the entire pH range because the pKa of chitosan is approximately 6.5^37^. Eamrat et al. (2024) found that using alum with chitosan at pH 5 resulted in a microplastic removal efficiency of 92.10%^31^. Ma et al. (2019) reported that removing PE MPs smaller than 0.5 μm. with aluminum-based coagulants at pH 7 achieved a treatment efficiency of 61%^19^. Raj et al. (2024) found that using ferric chloride with chitosan at pH 6.3 achieved a microplastic removal efficiency of 99.80%^25^. Huang et al. (2023) found that using polyaluminium chloride with chitosan is an efficient, stable, and pollution-free method for microplastic removal, with an efficiency of 90.00% due to improved charge neutralization and adsorption properties^27^. Generally, PAC is effective for removing colloidal particles in the pH range of 5–9, but its dissociation is less effective at a pH below 6^38^. This data also supports the findings of Azizi et al. (2023) who studied polyaluminium chloride, ferric chloride, and alum in solutions with particle sizes smaller than 500 microns and larger than 500 microns and found that a pH of 7 resulted in a microplastic removal efficiency of 81.00%^30^. as shown in Table 1.

**Table 1:** Summary of study results and literature review on the efficiency of coagulants and flocculants in microplastic removal.

Coagulant and coagulant aid	MPs	Size (mm)	pH*	RE** (%)	References
AlCl_3_⋅6H_2_O PAM	Polyethylene	< 0.5	7	45.34 ± 3.93	Ma et al.^19^
Al_2_(SO_4_)_3_ chitosan	Lowdensity polyethylene	< 0.090	5	92.10	Eamrat et al.^31^
Polyaluminium chloride	Polyethylene terephthalate	–	7	90.00	Huang et al.^27^
Polyaluminium chloride	Polyethylene, Polystyrene	< 0.5	7	29.70-77.83	Zhou et al.2^1^
Polyaluminium chloride	Polyethylene terephthalate	0.1–0.4	3–9	73.35	Zhang et al.^17^
FeCl_3_ chitosan	Polystyrene	0.0010	6.3	99.80	Raj et al.^25^
FeCl_3_	Polyethylene	< 0.5	6–8	61.00	Ma et al.^19^
FeCl_3_	Polyethylene	0.5-1	6–8	72.10	Arvaniti et al.^39^
Alum chitosan	Polyethylene	0.15	7	73.22	This study
Polyaluminium chloride chitosan	Polyethylene	0.15	7	77.41	This study
FeCl_3_ chitosan	Polyethylene	0.3	7	67.12	This study

*pH = potential of hydrogen, **RE = Removal Percentage.

### Study of microplastic removal efficiency of the filtration medium

**Fig. 4:**
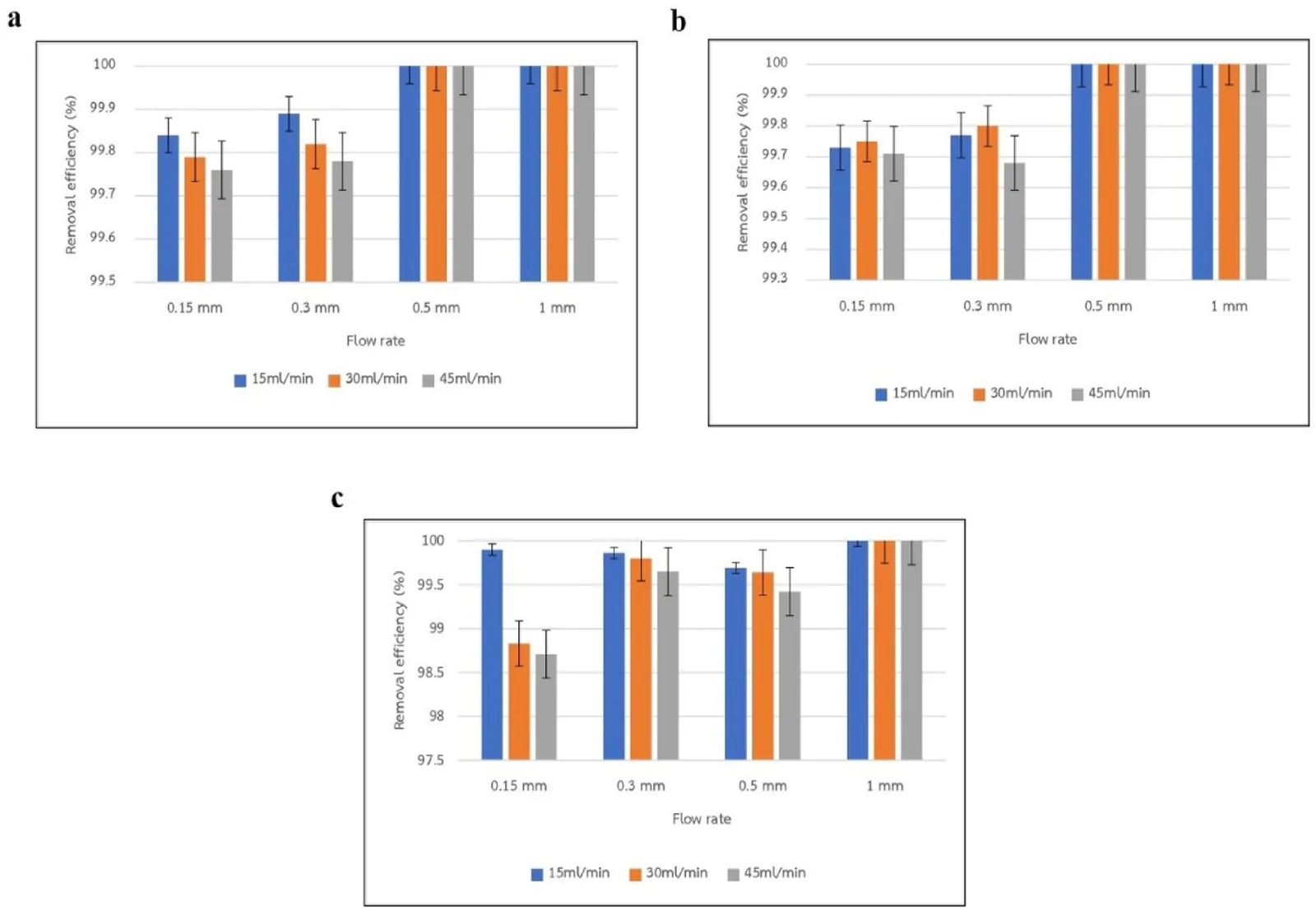
Microplastic removal efficiency of the filter media. (**a**) Condition1, (**b**) Condition2, (**c**) Condition 3.

The results of the study on the efficiency of the filtration medium for microplastic removal, divided into three experimental setups, showed that all three setups had similarly high microplastic removal efficiencies. Condition 1, at a flow rate of 15 mL/min, showed the highest microplastic filtration efficiency for sizes 0.15 mm and 0.3 mm, at 99.84% and 99.89%, respectively, and 100% for sizes 0.5 mm and 1 mm, as shown in Fig. 4a. Condition 2, at a flow rate of 30 mL/min, achieved the highest microplastic filtration efficiency for sizes 0.15 mm and 0.3 mm, at 99.75% and 99.80%, respectively, and 100% for sizes 0.5 mm and 1 mm, as shown in Fig. 4b. Condition 3, at a flow rate of 15 mL/min, achieved a maximum microplastic filtration efficiency of 98.90% for 0.15 mm, 99.86% for 0.3 mm, 99.69% for 0.5 mm, and 100% for 1 mm, as shown in Fig. 4c.

#### Study of microplastic removal efficiency of the filtration medium

The results indicated that all three experimental setups had similarly high microplastic removal efficiencies. Setup 1, with a flow rate of 15 mL/min, had the highest microplastic removal efficiency for sizes 0.15 mm and 0.3 mm (99.84% and 99.89%, respectively) and 100% for sizes 0.5 mm and 1 mm. In contrast, Setup 3, with a flow rate of 45 mL/min, had the lowest microplastic removal efficiency for sizes 0.15 mm, 0.3 mm, and 0.5 mm (98.71%, 99.68%, and 99.43%, respectively), while still achieving 100% for the 1 mm size. Based on the results, experimental setup 1 with a flow rate of 15 mL/min provided the highest overall removal efficiency at 99.93%. These findings support the concept from Chabi et al. (2023) that rapid sand filtration is highly effective (98.00%) at removing microplastics smaller than 10 micrometers from water, primarily through physical and chemical adsorption mechanisms^40^. According to the study by Sembiring, Fajar, & Handajani. (2021) on the removal efficiency of sand filters with treated water show that they can remove microplastics larger than 200 micrometers with 85–97% efficiency^41^. Similarly, Talvitie et al., 2017 found that rapid sand filters (RSF) achieved 97.00% microplastic removal from treated wastewater^33^. The study Twort et al. (2000) the RSF process relies on physical mechanisms for most particle removal. The porosity of the filter medium significantly affects microplastic removal efficiency^42^. Crittenden et al. (2012) explained that filtration occurs when the ratio of particle diameter to filter medium diameter exceeds 0.15 mm, meaning that smaller particles can pass through the medium^43^. The smallest effective size of sand filter medium is about 0.5 mm, which cannot trap particles smaller than 80 μm^43^. The results indicate that larger microplastics exhibit higher removal efficiencies through filtration processes. Sand is therefore an effective and low-cost filtration medium suitable for enhancing the efficiency of wastewater treatment systems prior to discharge into natural water bodies. However, this study was conducted only at the laboratory scale.

## Conclusion

### Type and quantity of coagulant

The use of alum, PAC, and FeCl_3_ as coagulants in conjunction with 20 mg/L of chitosan showed optimal dosages of 60 mg/L, 90 mg/L, and 90 mg/L, respectively. The pH value also significantly impacted removal, with the highest efficiency observed at a pH of 7. Therefore, the amount of coagulant, the amount of coagulant aid, and the pH all have a significant effect on microplastic removal.

### Microplastic size

Coagulation was most effective for removing small microplastics, specifically the 0.15 mm size. In contrast, the sand filter process was most effective at removing larger microplastics, specifically those greater than 0.5 mm.

### Enhancing microplastic removal efficiency

Adding a coagulant aid alongside a coagulant can significantly improve microplastic removal efficiency. The application of a sand filter column also showed that sand is an effective and low-cost filter for microplastics, making it suitable for future use in enhancing wastewater treatment systems.

### Recommendations for practical application

The selection of an appropriate coagulant, optimization of operating conditions, and the application of sand filtration may contribute to the reduction of microplastics in wastewater and could be considered for further evaluation in wastewater treatment systems.

### Limitations

Although this study showed high efficiency of sand filtration in removing microplastics, the size distribution of flocs prior to filtration was not fully characterized. Further investigation of floc size distribution could help improve the understanding of particle aggregation behavior, allow better comparison among different coagulants, and provide additional information for evaluating potential filter clogging and the practical performance of the filtration system^3,6^.

## Supplementary Information

Below is the link to the electronic supplementary material.


Supplementary Material 1


## Data Availability

The data that support the findings of this study are available from the article, or from the corresponding authors upon reasonable request.
