# Supplementary material for: Efficiency of a microplastic removal system from synthetic wastewater using a chemical process combined with simple filtration
Source: Sci Rep. 2026 Jun 20;16:27247. doi: 10.1038/s41598-026-59084-8 (PMC13529713; doi:10.1038/s41598-026-59084-8)
Supplement: Supplementary file 1 [file 41598_2026_59084_MOESM1_ESM.pdf]

Supplementary Materials

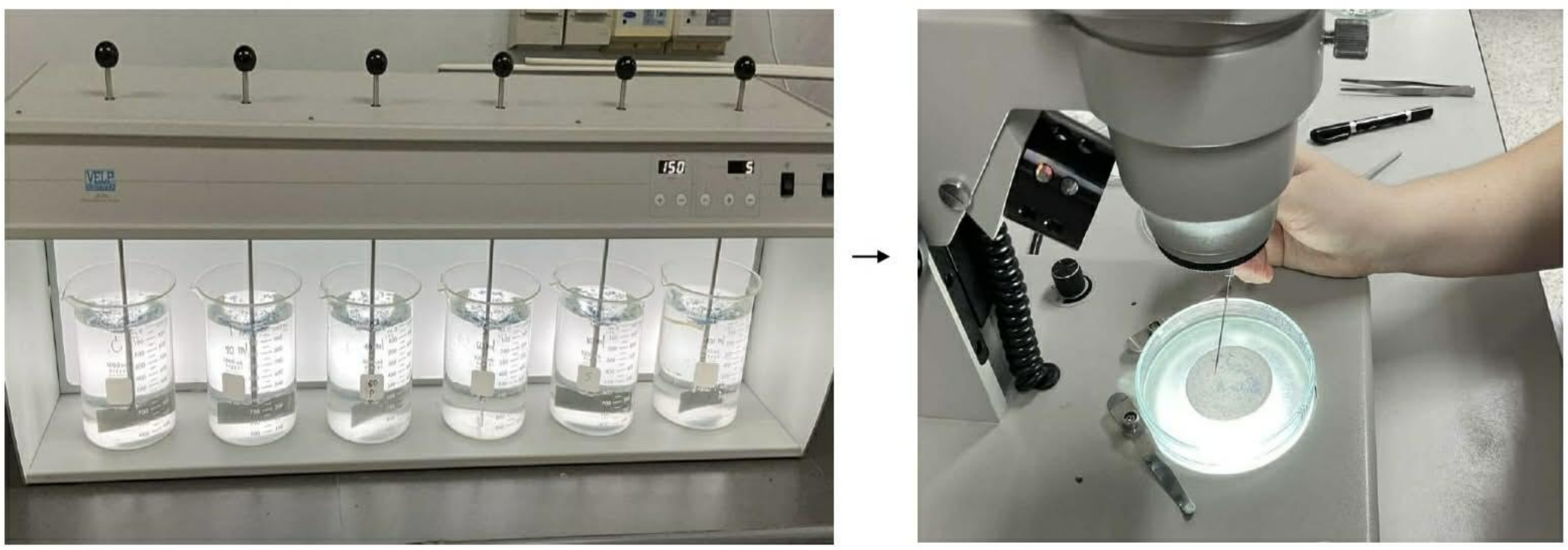

Coagulation experiment using the Jar Test

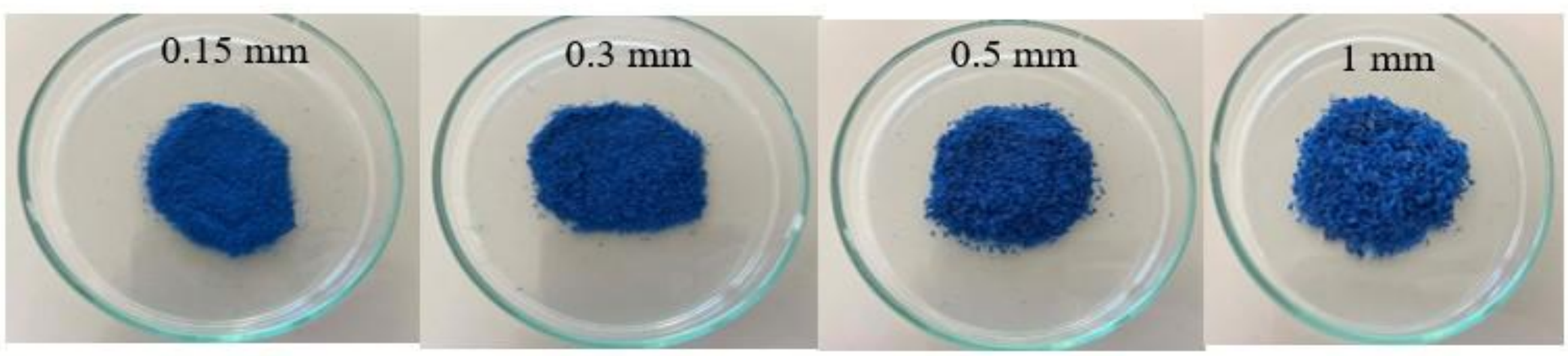

Sizes of microplastics used in the experiment
